# Production of Marine Probiotic Bacteria in a Cost-Effective Marine Media Based on Peptones Obtained from Discarded Fish By-Products

**DOI:** 10.3390/microorganisms8081121

**Published:** 2020-07-26

**Authors:** José Antonio Vázquez, Ana Durán, Margarita Nogueira, Araceli Menduíña, Joana Antunes, Ana Cristina Freitas, Ana María Gomes

**Affiliations:** 1 Grupo de Biotecnología y Bioprocesos Marinos, Instituto de Investigaciones Marinas (IIM-CSIC), C/Eduardo Cabello, 6, CP 36208 Vigo, Galicia, Spain; anais@iim.csic.es (A.D.); marga@iim.csic.es (M.N.); araceli@iim.csic.es (A.M.); 2 Laboratorio de Reciclado y Valorización de Materiales Residuales (REVAL), Instituto de Investigaciones Marinas (IIM-CSIC), C/Eduardo Cabello, 6, CP 36208 Vigo, Galicia, Spain; 3 Universidade Católica Portuguesa, CBQF—Centro de Biotecnologia e Química Fina—Laboratório Associado, Escola Superior de Biotecnologia, Rua Diogo Botelho 1327, 4169-005 Porto, Portugal; jantunes@porto.ucp.pt (J.A.); afreitas@porto.ucp.pt (A.C.F.); amgomes@porto.ucp.pt (A.M.G.)

**Keywords:** fish discards valorization, marine probiotics bacteria production, fish peptones, low-cost marine media, sustainability, logistic equation

## Abstract

The industrial production of marine bacteria with probiotic properties is limited by the excessive cost of the culture media adequate for their growth. The present work aimed to study the suitability of 30 marine media formulated with nitrogen sources (fish peptones) from different fish discards and seawater, for the growth of two marine probiotic bacteria (MPB), namely *Phaeobacter* sp. and *Pseudomonas fluorescens*. These fish peptones were produced from several discarded fish and by-products (heads, skins, and whole individuals of megrim, mackerel, gurnard, hake, etc.). In all cultivations, no significant differences were found on cell viability when the microorganism grew on commercial or alternative media. In relation to the biomass production, the growth of *Phaeobacter* sp. on waste media was commonly similar or a 20% lower than observed in the control cultures. For *P. fluorescens*, various peptones (skin peptones of pouting and blue whiting) showed even higher productive ability than commercial peptones. An initial economical evaluation revealed that low-cost media reduced until 120 times the cost of production of MPB.

## 1. Introduction

In the last two decades, intensive investigation dealing with the search and application of probiotic bacteria in aquaculture has been extensively reported [1,2,3,4]. The problems associated with the extensive mortalities produced in the first larvae stages of fish farming and the restrictive legislation in the use of antibiotics have demanded new alternatives based on, for instance, the use of lactic acid bacteria [5,6] or marine bacteria [7,8,9] with remarkable probiotic capacities against the lethal microorganisms present in pisciculture diseases. Different marine probiotic bacteria (MPB) belong to the *Proteobacteria* phylum as *Shewanella*, *Pseudomonas*, and *Phaeobacter* genera have shown excellent behaviors to improve the growth of fish cultures, increasing the activity of gastrointestinal microbiota and the immune responses of fish [10,11,12,13,14,15]. Among MPB, *Pseudomonas fluorescens* and *Phaeobacter* sp. have demonstrated skills of ecological competitiveness against fish pathogen bacteria [13,14,15,16] and they also enhanced larvae survival when they were added to turbot, oyster and salmon hatcheries [10,16,17,18]. Although the use of MPB has not yet been legalized in Europe, the need for solutions to avoid the application of antibiotics is increasing the pressure to commercialize effective bacteria to reduce fish farming mortalities.

In this context, the formulations of the media for the cultivation of bacteria are commonly rich in a source of organic nitrogen as free amino acids, protein hydrolysates, and/or peptones from different origins and commercial denominations: Bactopeptone, tryptone, meat extract, etc. One of the most specific broths is the marine medium (MM) especially designed for the culture of bacteria isolated from marine environments, and commonly employed in the growth of MPB [11,14]. It is composed of multiple mineral salts, simulating seawater, a low concentration of yeast extract, and a generic peptone.

Nevertheless, the application of these marine probiotics to aquaculture plants and hatcheries, when they can be marketed, will require the production of massive biomasses and viable cells at the industrial scale. However, marine medium and even the commercial peptones are too expensive, and the development of a cost-effective medium is fundamental for a continuous and sustainable supply of MPB. Fish wastes could be an interesting source of cheap and efficient peptones, the application of which has resulted in excellent growth yields of an extensive number of bacterial genera [19,20].

On the other hand, current European Union fishery policy is aimed at gradually eliminating fish discards. It forces fishing vessels to land all catches of regulated commercial species. The unwanted catches landed that cannot be directly sold for human consumption, due to the lack of a market, are considered as by-products [21]. As a result, the Landing Obligation (LO) policy will lead to an increasing amount of fish in European ports. While a fraction of this biomass may be suitable for the production of processed human food products, undersized and low-quality individuals require alternative plans [22,23]. Blue whiting, mackerel, megrim, gurnard, pouting, hake, Atlantic horse mackerel, hake, boarfish, and red scorpionfish are some of the most relevant, in tons, fish species landed in European ports but also the most discarded by fishing fleets [23], due to multiple motives: (a) Lack of quota, (b) species with low commercial value, or (c) being below legal sizes when are captured. One of the main challenges that faces the landing and valorize obligation imposed by the EU regulation No1380/2013, under the framework of the Common Fisheries Policy (CFP), is finding alternatives that help the fishery industry to overcome the costs associated with food-waste processing.

The present work investigated the potential of 30 peptones, obtained by enzymatic processing of whole individuals and head and skin by-products (obtained from fish mince production) of discarded fish generated from fishing activities, as a complex nitrogen source for the growth of *Phaeobacter* sp. and *P. fluorescens*. The kinetics of bacterial growth were perfectly modelled by a sigmoid equation formulated with parameters of clear biological meaning. Finally, a simple economical assessment was performed to corroborate the exceptional impact of the sustainable bioprocess proposed here.

## 2. Materials and Methods

### 2.1. Preparation of Peptones from Fish Discard By-Products

**Figure 1:**
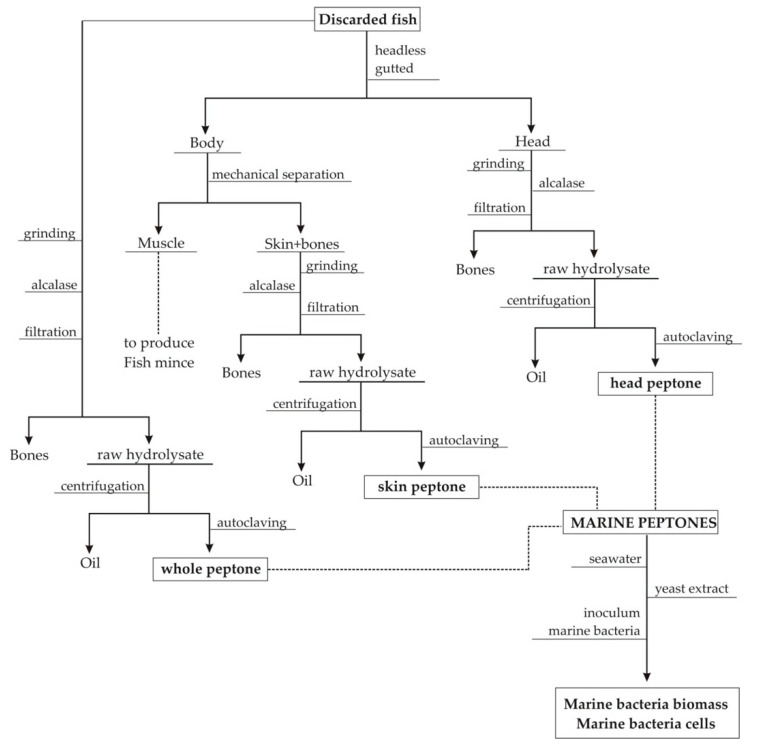
Flowchart of steps involved for the production of peptones from the skin, head, and whole individuals of discarded fish by fishing fleets.

The selected fish discards (captured in Atlantic North Ocean by Galician fishing fleets): grenadier (Gr, *Macrourus* sp.), megrim (Me, *Lepidorhombus boscii*), European hake (Ha, *Merluccius merluccius*), boarfish (Bo, *Capros aper*), Atlantic horse mackerel (AHM, *Trachurus trachurus*), blue whiting (BW, *Micromesistius poutassou*), mackerel (Ma, *Scomber scombrus*), red scorpionfish (RS, *Scorpaena scrofa*), pouting (Po, *Trisoreptus luscus*), and gurnard (Gu, *Trigla* spp.), were separated, on board, from commercial species and stored in ice. In port, the species were processed in the same day of landing. One portion of discarded fish was manually gutted and headed, and meat mince was mechanically separated from bones and skin using a bone separator (Baader 694, Lübeck, Germany). This fish mince was frozen in blocks for the later preparation of burgers, nuggets, and fingers [23,24]. The mixture of skins with bones (Sk), heads (He), and another portion of whole individuals (Wh) that were discarded was used as substrates for the production of fish peptones (Figure 1).

He, Sk, and Wh were ground in a meat grinder and stored at −18 °C. Enzymatic hydrolysis of these materials was performed in a controlled pH-Stat system with a 5-L glass-reactor (mixing 1 kg of milled discards with 2 L of distilled water, (S:L) ratio of 1:2 *w*/*v*). Alcalase 2.4 L (Novozymes, Nordisk, Bagsvaerd, Denmark) was the commercial protease applied at 1% (*v*/*w*), pH8.65, 200 rpm of agitation, and 60 °C for 4 h [25,26,27]. Bones were removed from raw hydrolysates by filtering and the liquid fraction was centrifuged (15,000× *g*/20 min) to separate oils and hydrolysates. The marine peptones were obtained after autoclaving (101 °C/60 min) and centrifuging these hydrolysates. A scheme of this procedure is shown in Figure 1. The basic composition of peptones is summarized in Table S1.

### 2.2. Microbiological Methods, Culture Media, and Analytical Determinations

The marine probiotic bacteria evaluated were *Phaeobacter* sp. DIFR 27−4 (Ph, called *Roseobacter* sp. before) and *Pseudomonas fluorescens* DIFR AH-2 (Pf). Ph and Pf were kindly provided by Dr. Lone Gram (DTU Aqua, Lyngby, Denmark). Stock cultures were stored at −80 °C in MM (Difco, Becton, Dickinson and Company, Sparks, MD, USA) with 25% glycerol. Inocula (1% *w*/*v*) consisted of cellular suspensions from 12–16 h cultures on MM. The composition of the culture media is shown in Table S2. The protein concentration in the media formulated with fish peptones was established by replacing the Lowry protein level in the peptone (2.6 g/L) present in MM. Mineral salts of this commercial broth were replaced by filtered and sterilized seawater. Thus, in alternative media, the fish peptones were mixed with seawater and 1 g/L of yeast extract (this was the only commercial ingredient added). Fermentations were carried out in triplicate using 300 mL Erlenmeyer flasks with 150 mL of medium at 22 °C and 200 rpm of orbital shaking. In all cases, the initial pH was adjusted to 7.5 with NaOH (5 N) and the media was sterilized separately at 121 °C for 15 min. The basic composition of fish peptones was determined in duplicate, as follows: (1) Reducing sugars (RS) by 3,5-dinitrosalicylic reaction [28], (2) total soluble proteins (Pr) using the Lowry method [29], and (3) total sugars (TS) by Dubois et al.’s protocol [30].

### 2.3. Bacterial Sampling and Biomass and Cell Analysis

Samples from each culture in alternative and control media were taken at predetermined times (3, 6, 9, 12, 16, 20, 24, and 30 h) and divided in two aliquots. The first aliquot was used for quantifying viable cells by means of a plate count technique on MM agar. Serial 10-fold dilutions were prepared in peptone-buffered solutions, and 0.1 mL samples were plated in triplicate, incubated at 22 °C for 48–72 h, and manually counted. For clarity, the results were expressed as G = ln(*N*/*N*_0_), where *N* is the colony-forming units per mL (cfu/mL) and *N*_0_ is the initial colony-forming units per mL (cfu/mL). The second aliquot was centrifuged at 3270× *g* for 15 min, from which the supernatant was used for determining the protein (Pr, Lowry method) consumption and the sediment was washed and resuspended in distilled water at an appropriate dilution to measure the optical density (OD) at 700 nm (A_700_). Then, the dry weight was estimated from a calibration curve (A_700_ vs. dry weight).

### 2.4. Mathematical Equations for Cultures Modelling

The growth of marine bacteria, biomass production (*X*), and cell formation (*G*) were predicted by the logistic equation [31]:

(1) $$P=\frac{P_{m}}{1+\exp\left[2+\frac{4v_{P}}{P_{m}}\left(\lambda_{P}-t\right)\right]}$$

Additionally, other parameters from Equation (1) were also calculated with the purpose of evaluating all the characteristics phases of the sigmoid growth [31]:

(2) $$\mu_{P}=\frac{4v_{P}}{P_{m}}$$

(3) $$\tau_{P}=\lambda_{P}+\frac{2}{\mu_{P}}$$

(4) $$t_{mP}=\tau_{P}+\frac{P_{m}}{2v_{P}}$$

where, *P* is the growth determined (*X* for biomass or *G* for viable cells); *t* is the time of culture (h); *P_m_* is the maximum growth (g/L for *X* and dimensionless for *G*); *v_P_* is the maximum growth rate (g L^−1^ h^−1^ for *X* and h^−1^ for *G*); *λ_P_* is the growth lag phase (h); *μ_P_* is the specific maximum growth rate (h^−1^); *τ_P_* is the time required to achieve half of the maximum growth (h); and *t_mP_* is the time required to reach the maximum growth (h). In addition, the yields of growth on protein uptakes (*Y_P_*/*Y_Pr_*) were also determined.

### 2.5. Economical Assessment of MPB Growth Costs

Based on the market prices of the MM ingredients and the values of *X_m_* and *G_m_* summarized in Table 1 and Table 2 and Tables S3 and S4, the costs of biomass (in €/g) and cells (in €/cell) production were calculated for the cultures with both bacteria in all media. In these calculations, the costs of the production of fish peptones (energy and reagents) were not included. These costs are highly dependent on the production scale of peptones (relatively higher on a smaller level of production) and difficult to determine on a laboratory scale. The present strategy of the valorization of heads and skins to produce fish peptones is a complementary alternative associated to the recovery of fish mince from fish discard biomasses that have to be landed under the LO normative. For a complete economical evaluation of such MPB, the process of fish mince production should also be incorporated in a further study.

### 2.6. Numerical and Statistical Analyses

Fitting procedures and parametric estimations calculated from the results were carried out by minimizing the sum of quadratic differences between the observed and model-predicted values, using the non-linear least-squares (quasi-Newton method) provided by the macro-‘Solver’ of the Microsoft Excel spreadsheet. Confidence intervals from the parametric estimates (Student’s *t* test) and the consistency of mathematical models (Fisher’s F test) and residual analysis (Durbin–Watson test) were evaluated by “SolverAid” macro (Levie’s Excellaneous website: http://www.bowdoin.edu/~rdelevie/excellaneous).

## 3. Results

Thirty fish peptones were produced by enzymatic hydrolysis of whole specimens of discarded fish and from by-products of these discards generated after fish mince recovery. They were prepared at a concentration of 2.6 g/L of Lowry protein (similarly to the standard level in MM) and the results were compared to the cultivations performed in commercial medium.

Table S1 summarizes the basic chemical composition of the fish peptones. The content of total soluble protein varied from 33 to 45 g/L for Sk, 28 to 40 g/L for He, and 36 to 54 g/L for Wh. The peptones also contained small amounts of total (0.45–1.61 g/L) and reducing sugars (0.09–0.45 g/L) due to the initial presence of fish muscle (tissue that accumulates a reserve of sugars) in the substrates employed for the production of such peptones. Taking into account that the new marine media included as maximum 10% of the final volume with fish peptones, the final concentrations of total and reducing sugars in these media were insignificant and always lower than 0.05 and 0.16 g/L, respectively.

**Figure 2:**
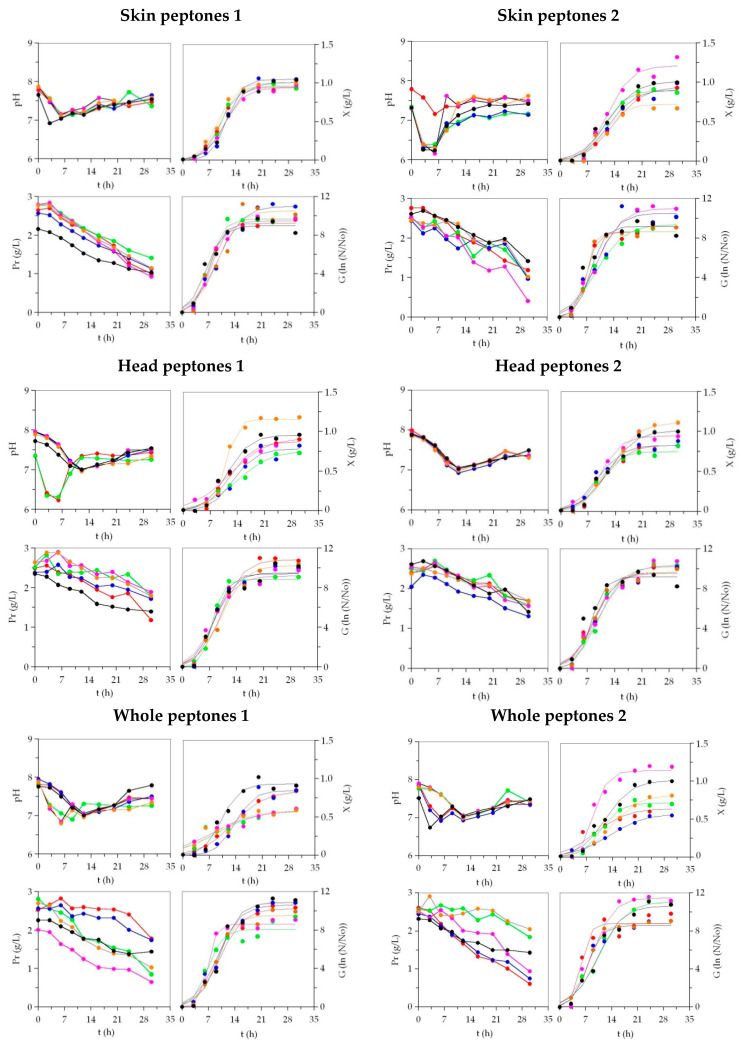
Culture kinetics of *Phaeobacter* sp. in marine media based on marine peptones from discarded fish and by-products. Peptones 1: ⯃: BW; ⯃: RS; ⯃: Ma; ⯃: Po; ⯃: Gu; ⯃: MM1. Peptones 2: ⯃: Gr; ⯃: Me; ⯃: Ha; ⯃: Bo; ⯃: AHM; ⯃: MM2. Experimental data of biomass (*X*) and viable cells (*G*) were fitted to the logistic equation. Protein (Pr) uptakes and pH are also shown. The confidence intervals of the experimental data (for two replicates) were in all cases less than 20% of the experimental mean values and omitted for clarity.

**Figure 3:**
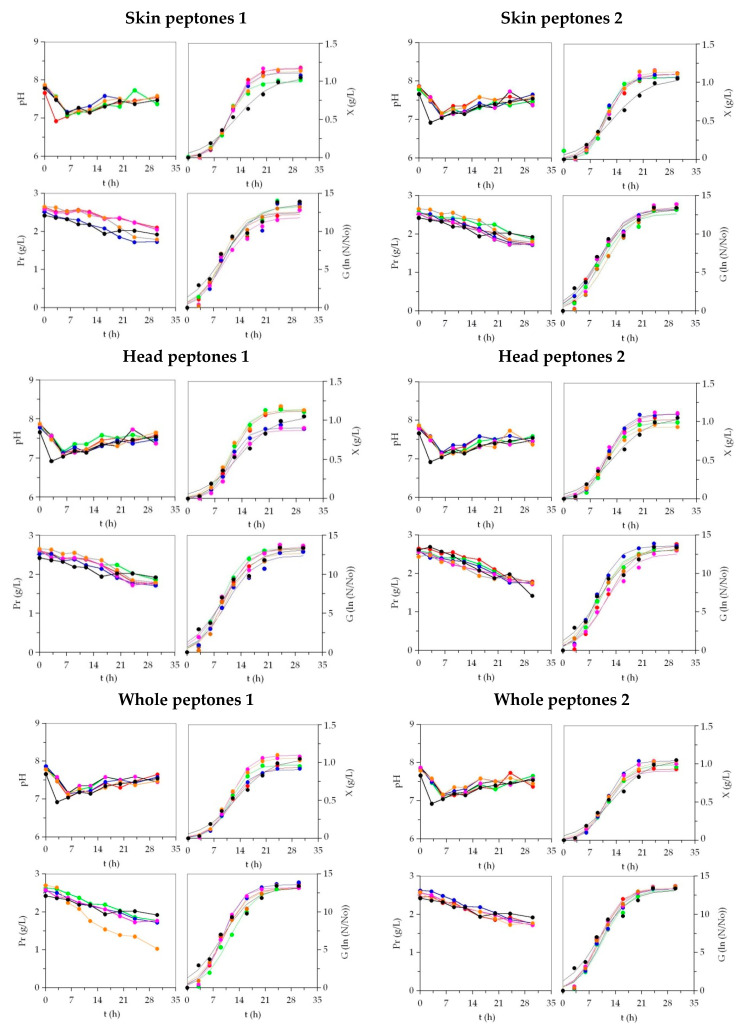
Culture kinetics of *P. fluorescens* in marine media based on marine peptones from discarded fish and by-products. Peptones 1: ⯃: BW; ⯃: RS; ⯃: Ma; ⯃: Po; ⯃: Gu; ⯃: MM1. Peptones 2: ⯃: Gr; ⯃: Me; ⯃: Ha; ⯃: Bo; ⯃: AHM; ⯃: MM2. Experimental data of biomass (*X*) and viable cells (*G*) were fitted to the logistic equation. Protein (Pr) uptakes and pH were also shown. The confidence intervals of experimental data (for two replicates) were in all cases less than 20% of the experimental mean values and omitted for clarity.

*Phaeobacter* sp. and *P. fluorescens* grew perfectly in all media tested (Figure 2 and Figure 3). The time-course of pH in the low-cost media generally followed similar patterns, with little exceptions, to those defined by MM. The experimental data of the growth, by optical density and plate-count, were in all cases mathematically modelled using the logistic Equation (1). The agreement between the experimental and predicted data was very good, with a goodness of fit that varied from 0.917 to 0.999 (Table 1 and Table 2 and Tables S3 and S4). In addition, the consistency of the logistic equation was corroborated for all the fittings by the F-Fisher test (*p* < 0.005). The numerical parameters from such an equation were statistically significant (t-Student test) except in 19 cases of 720, mainly lag-phases (*λ_x_* and *λ_G_*), where latencies were not clearly observed nor numerically identified.

In *Phaeobacter* sp. cultures (Table 1 and Table 2), higher *X_m_* values were mostly obtained in peptones of Sk than in peptones of He and Wh, but they were similar to the control outcomes (with exception of SK_AHM). The lowest maximum biomass production was found in the peptones of Wh. Nevertheless, the largest values were reached in Sk_Bo (1.21 g/L), He_Gu (1.16 g/L), and Wh_Bo (1.14 g/L), the first two being significantly more productive than MM (*p* < 0.05). The growth quantified by viable cell production followed a similar response in alternative peptones: The Sk origin was slightly most valuable than He and Wh, and the best options were Wh_Bo, Sk_Bo, and Sk_Gu. The growth rate parameters (*v_x_*, *v_G_*, *μ_x_* and *μ_G_*) were higher in some discard-based peptones than in MM, but the differences were not significant (*p* > 0.05). These findings were in agreement with the time-dependent parameters (*λ_x_*, *λ_G_*, *t_mx_*, *t_mG_*, *τ_x_*, and *τ_G_*) that show similar statistical values between all media.

The maximum biomass production of *P. fluorescens* in media containing Sk_peptones was also higher than in the rest of the peptones. Peptones from the skins of BW and Po revealed the best results whereas peptones from the heads of Bo and Gr led to the lowest dry weights. The numerical data in both pairs of peptones were statistically different in comparison to that predicted for MM (*p* > 0.05). Faster biomass production (*v_x_* and *μ_x_*) and shorter time-dependent parameters (*t_mX_* and *τx*) were reached in MM (Tables S3 and S4). Nevertheless, the improvements of the kinetic parameters in the commercial medium compared with most of the low-cost media were not significant. The results of viable cell growth for *P. fluorescens* in the different media were visually almost indistinguishable and parametrically similar between cultures. No significant differences between media were found for all the parameters that characterize such growth determinations.

The production yields for *Phaeobacter* biomass (*Y_X_/Y_Pr_*) were globally larger in He_peptones than Sk_peptones, Wh_peptones, and MM, but the best yield was found in Wh_AHM. However, for Pf, those ratios of biomass production as a function of the protein uptake showed larger values in Sk_peptones, with BW, Po, and Me being the most efficient substrates. In the case of viable cells of MPB (*Y_G_/Y_Pr_*), the generic trends were similar to those described for *Y_X_/Y_Pr_*: He_peptones and Sk_peptones as the best productive source of organic nitrogen for *Phaeobacter* sp. and *P. fluorescens*, respectively. Sk_Me, SK_BW, and Sk_Po in *Phaeobacter* sp.and Sk_Gu in *P. fluorescens* were the most efficient media in the consumption of proteins.

Table S5 shows the costs of the production of MPB biomass and viable cells in the new marine media (based on fish peptones and seawater) and in the corresponding commercial MM. Compared against the control, the decrease of biomass and cell growth costs for *Phaeobacter* sp. ranged from 68–120 times and 81–107-fold, respectively, depending on the fish peptone used (with a higher reduction in Sk_Bo and He_Gu). In the case of *P. fluorescens*, these reductions in relation to MM production were in the interval 83–111 times for *X_m_* and 88–101 times for *G_m_* (with a lower decrease in He_Bo and He_Gr).

## 4. Discussion

In the current study we completed the total valorization of wastes (heads and skins with bones) generated in the previous manual and mechanical separation of fish mince useful for the elaboration of human foods [24]. This sustainable strategy was also extended to the whole individuals of the same discarded species, which, in some situations, are not allowed to be introduced in the preparation of products for human consumption [23].

Our results indicated that the nutritive formulations based on the mixture of peptones obtained from fish discards’ hydrolysis and seawater are cost-effective alternatives to produce marine probiotic bacteria at a large scale. Most of them were perfect substitutes of commercial marine medium for both bacteria studied. The maximum production of biomass and cells by Ph on the cited media was similar to those obtained in media containing peptones extracted from yellowfin tuna wastes and more than 20% higher than the production found using rainbow trout, swordfish, and squid by-products [32,33]. In the case of Pf, the present results also improved the production observed in those referenced organic nitrogen sources [32,33]: The values of maximum biomass in most of the alternative media (such as those formulated here with Sk_BW, Sk_Po, Sk_Gu, and He_Po) were 30% superior to the best results reported for peptones from squid and trout viscera. Additionally, tuna and trout waste materials were demonstrated to be an adequate source of nitrogen for the production of alkaline proteases by two *Vibrio* species [34]. This genus and the metabolites were not studied here and could be an interesting potential application of peptones from discarded fish in further works.

In terms of processing, the recovery of protein sources from fish wastes by enzyme proteolysis is a well-documented alternative [35,36]. One of the cheapest approaches is using the endogenous enzymes present in the pancreas or pyloric cecum (autohydrolysis step) when fish viscera residues are valorized [37,38,39]. The peptones thus obtained have been useful for the production of bacteriocins (nisin and pediocin) by *Lactococcus lactis* and *Pediococcus acidilactici* [37,40]. However, the difficulty in controlling the process of biocatalysis with the corresponding variability on the kind and size of peptides generated advises the application of exogenous enzymes. Between commercial proteases, alcalase (an endoprotease) is one of the most valuable for the digestion of fully fish residues to yield high degrees of hydrolysis and in vitro digestibility, and to tailor the molecular weight of the peptides [32,36,41,42,43]. For instance, hydrolysates of tuna head and cod viscera obtained by alcalase were adequate for lactic acid bacteria production [19,44]. Nevertheless, few studies of marine bacteria on fish protein hydrolysates have been conducted. Biomasses of *Vibrio anguillarum*, *Roseobacter* sp., *Carnobacterium divergens*, and *Aeromonas salmonicida* were successfully produced in peptones from fish by-products and cephalopod effluents [20,37,45]. The low-cost marine media developed here, including filtered seawater, yeast extract, and peptones from hydrolysates of discarded species and by-products, has been unexplored to date.

Finally, we must indicate that the proposed evaluation of cultures by means of the kinetic parameters obtained from Equation (1) was in all cases successful since the experimental data were perfectly simulated and the parameters defined all the sigmoid growth phases [31]. The logistic equation is a well-known mathematical resource extensively utilized in different fields, such as animal growth [46,47], predictive microbiology [48], microbial metabolite productions [49], and analysis of DNA sequencing [50], among others. The present economic decreasing production costs for biomass and cell formation of MPB were in agreement with values previously reported for protein-rich effluents from chitin purification [32]. The very low cost of MPB growth on alternative marine media may suppose a valuable tool for the sustainable management of discarded fish residues and for the massive production of viable probiotics adequate for fighting infections in aquaculture fish diseases.

## 5. Conclusions

This work showed that a cost-effective media formulated with peptones obtained by the enzyme hydrolysis of fish discards (head, skins, whole individuals), filtered seawater, and a very low concentration of yeast extract can be an excellent substitute of the commercial marine medium to produce marine probiotic bacteria, such as *Phaeobacter* sp. and *Pseudomonas fluorescens*. For both bacteria, viable cellular productions were almost always, at least, similar to those found in MM. The biomass kinetics (quantified as dry weight) of Pf and Ph showed similar or slight differences between the low-cost and control media. In economic terms, the alternative media led to a huge reduction of marine probiotic bacteria growth costs, between 68- and 12-fold depending on the strain and variable evaluated, in comparison with those found in commercial MM.

## Figures and Tables

**Table 1:** Numerical values and confidence intervals for parameters obtained from experimental data of *Phaeobacter* sp. (Ph) growth modelled by a logistic equation (1-4). R^2^ is the determination coefficient among the experimental and predicted data. The production yields (*Y_X/_Y_Pr_* and *Y_G/_Y_Pr_*) are also shown. NS: not significant. MM1 and MM2 were the controls kinetics in commercial marine medium (MM) performed for each set of cultures. Consistency of fittings was also determined (*p*-value from F-Fisher test).

Biomass (X)																
	Sk_BW	Sk_RS	Sk_Ma	Sk_Po	Sk_Gu	Sk_Gr	Sk_Me	Sk_Ha	Sk_Bo	Sk_AHM	He_BW	He_RS	He_Ma	He_Po	He_Gu	MM1
***X_m_***	0.95 ± 0.04	0.94 ± 0.04	0.96 ± 0.04	0.95 ± 0.05	1.05 ± 0.04	1.00 ± 0.05	0.90 ± 0.08	0.91 ± 0.15	1.21 ± 0.15	0.72 ± 0.14	0.87 ± 0.09	0.77 ± 0.12	0.78 ± 0.13	0.92 ± 0.13	1.16 ± 0.06	1.01 ± 0.05
***v_m_***	0.10 ± 0.02	0.09 ± 0.02	0.12 ± 0.02	0.06 ± 0.01	0.13 ± 0.03	0.11 ± 0.03	0.08 ± 0.03	0.07 ± 0.05	0.10 ± 0.05	0.07 ± 0.06	0.07 ± 0.03	0.04 ± 0.01	0.07 ± 0.04	0.05 ± 0.01	0.19 ± 0.06	0.10 ± 0.02
***λ_X_***	5.27 ± 0.99	5.83 ± 1.02	5.91 ± 0.89	6.43 ± 1.02	7.66 ± 0.82	4.61 ± 1.22	5.88 ± 1.98	5.79 ± 3.98	6.40 ± 2.67	6.16 ± 4.37	5.97 ± 2.27	5.03 ± 2.69	7.17 ± 3.29	2.50 (NS)	7.81 ± 0.89	6.19 ± 1.73
***μ_X_***	0.44 ± 0.09	0.38 ± 0.08	0.49 ± 0.10	0.27 ± 0.04	0.51 ± 0.11	0.42 ± 0.11	0.37 ± 0.14	0.30 ± 0.18	0.34 ± 0.17	0.39 ± 0.35	0.33 ± 0.13	0.23 ± 0.09	0.48 (NS)	0.21 ± 0.08	0.65 ± 0.24	0.38 ± 0.13
***τ_X_***	9.86 ± 0.56	11.0 ± 0.6	9.98 ± 0.49	13.8 ± 0.8	11.6 ± 0.5	9.37 ± 0.69	11.3 ± 1.3	12.4 ± 2.4	12.3 ± 1.8	11.3 ± 2.7	12.1 ± 1.5	13.9 ± 2.4	13.1 ± 3.5	11.9 ± 2.3	10.9 ± 0.4	11.1 ± 1.1
***t_mX_***	14.5 ± 1.3	16.3 ± 1.4	14.1 ± 1.1	21.1 ± 1.7	15.6 ± 1.1	14.1 ± 1.5	16.7 ± 2.8	18.9 ± 5.5	18.2 ± 4.0	16.4 ± 6.1	18.2 ± 3.4	22.8 ± 5.2	18.9 ± 4.9	21.4 ± 5.3	13.9 ± 1.1	16.7 ± 2.4
***Y_X_/Y_Pr_***	0.564	0.522	0.674	0.592	0.729	0.615	0.582	0.669	0.616	0.468	0.682	1.130	1.228	1.273	1.362	0.920
***R*^2^**	0.996	0.997	0.997	0.998	0.988	0.995	0.989	0.970	0.981	0.943	0.987	0.984	0.968	0.983	0.998	0.990
***p*-value**	<0.005	<0.005	<0.005	<0.005	<0.005	<0.005	<0.005	<0.005	<0.005	<0.005	<0.005	<0.005	<0.005	<0.005	<0.005	<0.005
**Cells (G)**																
	**Sk_BW**	**Sk_RS**	**Sk_Ma**	**Sk_Po**	**Sk_Gu**	**Sk_Gr**	**Sk_Me**	**Sk_Ha**	**Sk_Bo**	**Sk_AHM**	**He_BW**	**He_RS**	**He_Ma**	**He_Po**	**He_Gu**	**MM1**
***G_m_***	9.23 ± 0.44	9.48 ± 0.69	9.68 ± 0.72	10.2 ± 1.2	11.1 ± 1.5	10.2 ± 0.8	9.40 ± 1.27	10.5 ± 1.3	11.0 ± 1.2	8.62 ± 0.39	10.8 ± 1.3	8.84 ± 0.34	9.40 ± 0.86	9.17 ± 1.09	10.4 ± 1.6	10.5 ± 2.2
***v_G_***	1.24 ± 0.33	1.13 ± 0.43	1.27 ± 0.52	1.14 ± 0.34	0.79 ± 0.33	1.49 ± 0.58	0.67 ± 0.30	0.94 ± 0.63	0.86 ± 0.35	2.00 ± 0.73	0.95 ± 0.43	1.61 ± 0.39	1.12 ± 0.53	0.82 ± 0.42	0.86 ± 0.41	1.03 ± 0.44
***λ_G_***	3.54 ± 1.13	3.58 ± 1.77	3.51 ± 1.77	3.86 ± 1.62	3.78 ± 3.04	6.87 ± 1.57	2.54 (NS)	3.72 (NS)	3.17 ± 2.75	4.69 ± 0.79	4.41 ± 2.76	5.04 ± 0.78	3.96 ± 2.21	2.48 (NS)	3.72 ± 3.30	3.37 ± 2.54
***μ_G_***	0.54 ± 0.15	0.48 ± 0.19	0.53 ± 0.23	0.41 ± 0.13	0.29 ± 0.14	0.53 ± 0.22	0.28 ± 0.15	0.36 ± 0.26	0.31 ± 0.17	0.93 ± 0.35	0.35 ± 0.18	0.73 ± 0.18	0.48 ± 0.24	0.36 ± 0.20	0.30 ± 0.16	0.36 ± 0.17
***τ_G_***	7.27 ± 0.62	7.76 ± 0.97	7.32 ± 0.96	8.79 ± 0.91	10.8 ± 2.0	10.6 ± 0.9	9.59 ± 2.12	9.35 ± 2.39	9.59 ± 1.69	6.84 ± 0.49	10.1 ± 1.7	7.79 ± 0.43	8.20 ± 1.21	8.07 ± 1.76	10.4 ± 2.1	8.93 ± 1.47
***t_mG_***	11.0 ± 1.3	12.0 ± 2.1	11.1 ± 2.0	13.7 ± 2.0	17.8 ± 4.6	14.4 ± 1.9	16.6 ± 4.9	15.1 ± 5.4	16.0 ± 3.9	9.00 ± 1.08	15.9 ± 5.2	10.5 ± 0.8	12.4 ± 2.6	13.7 ± 3.9	17.0 ± 4.9	14.5 ± 3.3
***Y_G_/Y_Pr_***	4.71	4.83	3.70	5.989	24.13	8.449	6.81	5.989	14.54	6.37	14.74	12.80	15.13	13.05	6.500	13.63
***R*^2^**	0.994	0.988	0.985	0.991	0.977	0.990	0.973	0.949	0.979	0.994	0.977	0.996	0.981	0.971	0.969	0.981
***p*-value**	<0.005	<0.005	<0.005	<0.005	<0.005	<0.005	<0.005	<0.005	<0.005	<0.005	<0.005	<0.005	<0.005	<0.005	<0.005	<0.005

**Table 2:** Continuation of Table 1.

Biomass (X)																
	He_Gr	He_Bo	He_Ha	He_AHM	He_Me	Wh_BW	Wh_RS	Wh_Ma	Wh_Po	Wh_Gu	Wh_Gr	Wh_Bo	Wh_Ha	Wh_Me	Wh_AHM	MM2
***X_m_***	0.83 ± 0.11	0.94 ± 0.09	0.83 ± 0.10	1.11 ± 0.08	0.75 ± 0.07	0.85 ± 0.05	0.69 ± 0.20	0.85 ± 0.11	0.68 ± 0.19	0.69 ± 0.17	0.74 ± 0.16	1.14 ± 0.20	0.68 ± 0.05	0.71 ± 0.07	0.82 ± 0.04	0.95 ± 0.10
***v_m_***	0.07 ± 0.03	0.09 ± 0.04	0.07 ± 0.04	0.08 ± 0.02	0.08 ± 0.02	0.05 ± 0.01	0.04 ± 0.02	0.09 ± 0.04	0.04 ± 0.02	0.05 ± 0.02	0.06 ± 0.03	0.15 ± 0.11	0.05 ± 0.01	0.07 ± 0.03	0.06 ± 0.01	0.08 ± 0.03
***λ_X_***	4.55 ± 3.07	4.89 ± 2.36	3.10 ± 3.09	6.04 ± 1.49	5.00 ± 2.11	4.90 ± 1.15	−2.1 (NS)	8.81 ± 2.54	−3.9 (NS)	−1.6 (NS)	0.75 (NS)	5.27 ± 2.32	3.47 ± 1.63	5.80 ± 2.32	6.42 ± 1.01	5.72 ± 2.26
***μ_X_***	0.31 ± 0.16	0.38 ± 0.17	0.32 ± 0.16	0.29 ± 0.07	0.42 ± 0.18	0.24 ± 0.04	0.18 ± 0.17	0.40 ± 0.21	0.17 ± 0.17	0.20 ± 0.19	0.24 ± 0.20	0.54 ± 0.19	0.21 ± 0.05	0.40 ± 0.19	0.27 ± 0.04	0.35 ± 0.14
***τ_X_***	10.9 ± 2.0	10.2 ± 1.4	9.38 ± 1.87	12.9 ± 1.1	9.82 ± 1.20	13.4 ± 1.0	9.06 ± 6.16	13.8 ± 1.7	7.65 ± 6.00	8.23 ± 5.07	9.25 ± 4.19	8.99 ± 1.40	13.1 ± 1.5	10.8 ± 1.4	13.8 ± 0.8	11.5 ± 1.5
***t_mX_***	17.3 ± 4.5	15.5 ± 3.2	15.7 ± 4.3	20.1 ± 2.6	14.6 ± 2.7	21.9 ± 2.1	20.3 ± 15.3	18.8 ± 3.6	19.2 ± 15.7	18.0 ± 12.6	17.8 ± 9.9	12.7 ± 3.1	22.8 ± 3.3	15.9 ± 3.1	21.1 ± 1.7	17.3 ± 3.3
***Y_X_/Y_Pr_***	1.225	0.970	1.196	1.639	0.884	1.103	0.463	1.009	0.588	0.653	0.547	0.899	0.463	0.955	1.749	1.006
***R*^2^**	0.975	0.981	0.973	0.995	0.985	0.997	0.924	0.979	0.925	0.924	0.917	0.993	0.994	0.983	0.998	0.986
***p*-value**	<0.005	<0.005	<0.005	<0.005	<0.005	<0.005	<0.05	<0.005	<0.05	<0.05	<0.01	<0.005	<0.005	<0.005	<0.005	<0.005
***Cells (G)***																
	**He_Gr**	**He_Bo**	**He_Ha**	**He_AHM**	**He_Me**	**Wh_BW**	**Wh_RS**	**Wh_Ma**	**Wh_Po**	**Wh_Gu**	**Wh_Gr**	**Wh_Bo**	**Wh_Ha**	**Wh_Me**	**Wh_AHM**	**MM2**
***G_m_***	9.56 ± 1.09	10.4 ± 1.5	9.67 ± 1.06	10.6 ± 0.9	8.62 ± 0.48	9.20 ± 1.00	8.09 ± 1.16	10.6 ± 1.1	8.82 ± 1.02	9.52 ± 0.73	10.1 ± 1.1	11.4 ± 1.3	8.57 ± 0.70	8.78 ± 0.33	9.39 ± 0.58	9.99 ± 2.18
***v_G_***	0.85 ± 0.41	0.74 ± 0.35	0.87 ± 0.40	0.94 ± 0.38	1.54 ± 0.58	0.86 ± 0.32	1.04 ± 0.82	0.90 ± 0.36	1.77 ± 1.77	0.99 ± 0.35	0.92 ± 0.36	0.81 ± 0.33	1.13 ± 0.51	1.13 ± 0.23	1.09 ± 0.34	0.94 ± 0.53
***λ_G_***	3.00 ± 2.92	3.04 (NS)	3.27 ± 2.77	3.49 ± 2.26	4.06 ± 1.16	4.11 ± 2.36	2.97 (NS)	3.54 ± 2.55	3.31 ± 2.81	4.22 ± 1.87	4.43 ± 2.40	3.80 ± 2.80	3.63 ± 1.93	3.54 ± 0.89	3.04 ± 1.53	3.26 ± 3.13
***μ_G_***	0.36 ± 0.19	0.29 ± 0.16	0.36 ± 0.18	0.39 ± 0.17	0.71 ± 0.28	0.33 ± 0.14	0.51 ± 0.43	0.34 ± 0.15	5.80 ± 1.27	0.42 ± 0.16	0.34 ± 0.15	0.30 ± 0.14	0.53 ± 0.25	0.52 ± 0.11	0.46 ± 0.15	0.39 ± 0.24
***τ_G_***	8.61 ± 1.68	10.0 ± 2.2	8.83 ± 1.60	8.61 ± 1.27	6.86 ± 0.64	10.1 ± 1.5	6.88 ± 1.90	9.47 ± 1.52	11.0 (NS)	9.02 ± 1.05	10.3 ± 1.5	10.4 ± 1.8	7.42 ± 1.05	7.41 ± 0.49	7.36 ± 0.84	8.33 ± 1.76
***t_mG_***	14.2 ± 3.8	17.0 ± 5.2	14.4 ± 3.6	13.8 ± 2.8	9.67 ± 1.36	16.1 ± 3.3	10.8 ± 4.0	15.4 ± 3.5	8.29 ± 2.90	13.8 ± 2.3	16.2 ± 3.3	17.0 ± 4.1	11.2 ± 2.2	11.3 ± 1.0	11.7 ± 1.8	13.4 ± 3.9
***Y_G_/Y_Pr_***	14.12	11.21	13.91	14.85	4.27	11.31	5.10	13.00	4.44	11.71	5.273	6.77	6.20	6.95	4.67	10.67
***R*^2^**	0.975	0.971	0.976	0.983	0.991	0.984	0.946	0.981	0.956	0.988	0.982	0.979	0.983	0.996	0.990	0.968
***p*-value**	<0.005	<0.005	<0.005	<0.005	<0.005	<0.005	<0.005	<0.005	<0.005	<0.005	<0.005	<0.005	<0.005	<0.005	<0.005	<0.005

## Supplementary Materials

The following are available online at https://www.mdpi.com/2076-2607/8/8/1121/s1, Table S1: Basic biochemical composition of fish peptones, Table S2: Composition of culture media for marine probiotic bacteria, Table S3: Parameter kinetics of Pf cultures, Table S4: Continuation of Table S3, Table S5: Cost of production of biomass and bacterial cells.

## References

[1] Vine N.G., Leukes W.D., Kaiser H. (2006). Probiotics in marine larviculture. FEMS Microbiol. Rev..

[2] Defoirdt T., Boon N., Sorgeloos P., Verstraete W., Bossier P. (2007). Alternatives to antibiotics to control bacterial infections: Luminescent vibriosis in aquaculture as an example. Trends Biotechnol..

[3] Wang Y.-B., Li J.-R., Lin J. (2008). Probiotics in aquaculture: Challenges and outlook. Aquaculture.

[4] Rasmussen B.B., Kalatzis P.G., Middelboe M., Gram L. (2019). Combining probiotic *Phaeobacter inhibens* DSM17395 and broad-host-range vibriophage KVP40 against fish pathogenic vibrios. Aquaculture.

[5] Planas M., Vázquez J.A., Marques J., Pérez-Lomba R., González M.P., Murado M.A. (2004). Enhancement of rotifer (*Brachionus plicatilis*) growth by using terrestrial acid lactic bacteria. Aquaculture.

[6] Dash G., Raman R.P., Prasad K.P., Makesh M., Pradeep M.A., Sen S. (2015). Evaluation of paraprobiotic applicability of *Lactobacillus plantarum* in improving the immune response and disease protection in giant freshwater prawn, *Macrobrachium rosenbergii* (de Man, 1879). Fish Shellfish Immunol..

[7] Pintado J., Pérez-Lorenzo M., Luna-González A., Sotelo C.G., Prol M.J., Planas M. (2010). Monitoring of the bioencapsulation of a probiotic *Phaeobacter* strain in the rotifer *Brachionus plicatilis* using denaturing gradient gel electrophoresis. Aquaculture.

[8] D’Alvise P.W., Lillebø S., Prol-Garcia M.J., Wergeland H.I., Nielsen K.F., Bergh Ø., Gram L. (2012). *Phaeobacter gallaeciensis* reduces *Vibrio anguillarum* in cultures of microalgae and rotifers, and prevents vibriosis in cod larvae. PLoS ONE.

[9] Grotkjær T., Bentzon-Tilia M., D’Alvise P., Dourala N., Nielsen K.F., Gram L. (2016). Isolation of TDA-producing *Phaeobacter* strains from sea bass larval rearing units and their probiotic effect against pathogenic *Vibrio* spp. in Artemia cultures. Syst. Appl. Microbiol..

[10] Planas M., Pérez-Lorenzo M., Hjelm M., Gram L., Fiksdal I.U., Bergh Ø., Pintado J. (2006). Probiotic effect In Vivo of *Roseobacter* strain 27-4 against *Vibrio* (*Listonella*) *anguillarum* infections in turbot (*Scophthalmus maximus* L.) larvae. Aquaculture.

[11] D’Alvise P.W., Lillebø S., Wergeland H.I., Gram L., Bergh Ø. (2013). Protection of cod larvae from vibriosis by *Phaeobacter* spp.: A comparison of strains and introduction times. Aquaculture.

[12] Prol-García M.J., Pintado J. (2013). Effectiveness of probiotic *Phaeobacter* bacteria grown in biofilters against *Vibrio anguillarum* infections in the rearing of turbot (*Psetta maxima*) Larvae. Mar. Biotechnol..

[13] Gram L., Løvold T., Nielsen J., Melchiorsen J., Spanggaard B. (2001). In Vitro antagonism of the probiont *Pseudomonas fluorescens* strain AH2 against *Aeromonas salmonicida* does not confer protection of salmon against furunculosis. Aquaculture.

[14] D’Alvise P.W., Melchiorsen J., Porsby C.H., Nielsen K.F., Gram L. (2010). Inactivation of *Vibrio anguillarum* by attached and planktonic roseobacter cells. Appl. Environ. Microbiol..

[15] Prol-García M.J., Gómez M., Sánchez L., Pintado J. (2014). *Phaeobacter* grown in biofilters: A new strategy for the control of Vibrionaceae in aquaculture. Aquac. Res..

[16] Gram L., Melchiorsen J., Spanggaard B., Huber I., Nielsen T.F. (1999). Inhibition of *Vibrio anguillarum* by *Pseudomonas fluorescens* AH2, a possible probiotic treatment of fish. Appl. Environ. Microbiol..

[17] Porsby C.H., Gram L. (2016). *Phaeobacter inhibens* as biocontrol agent against *Vibrio vulnificus* in oyster models. Food Microbiol..

[18] Sonnenschein E.C., Phippen C.B.W., Nielsen K.F., Mateiu R.V., Melchiorsen J., Gram L., Overmann J., Freese H.M. (2017). *Phaeobacter piscinae* sp. nov., a species of the Roseobacter group and potential aquaculture probiont. Int. J. Syst. Evol. Microbiol..

[19] Safari R., Motamedzadegan A., Ovissipour M., Regenstein J.M., Gildberg A., Rasco B. (2012). Use of hydrolysates from yellowfin tuna (*Thunnus albacares*) heads as a complex nitrogen. Food Bioprocess Technol..

[20] Gildberg A., Dahl R., Mikkelsen H., Nilsen K. (2010). Peptones from Atlantic cod stomach as nitrogen sources in growth media to marine bacteria. J. Aquat. Food Prod. Technol..

[21] European Commission (2020). Discarding and the Landing Obligation. https://ec.europa.eu/fisheries/cfp/fishing_rules/discards_en.

[22] Iñarra B., Bald C., Cebrián M., Antelo L.T., Franco-Uría A., Vázquez J.A., Pérez-Martín R.I., Zufía J., Uhlmann S.S., Ulrich C., Kennelly S.J. (2019). What to do with unwanted catches: Valorisation options and selection strategies. The European Landing Obligation, Reducing Discards in Complex, Multi-Species and Multi-Jurisdictional Fisheries.

[23] Pérez-Martín R.I., Antelo L.T., Vázquez J.A., Mirón J. (2020). An on-land management and valorisation approach for biomass associated with landing obligation compliance. Mar. Policy.

[24] Borderías A.J., Moreno H.M. (2018). Valorization of recurrently discarded fish species in trawler fisheries in North-West Spain. J. Food Sci. Technol..

[25] Vázquez J.A., Fernández-Compás A., Blanco M., Rodríguez-Amado I., Moreno H., Borderías J., Pérez-Martín R.I. (2019). Development of bioprocesses for the integral valorisation of fish discards. Biochem. Eng. J..

[26] Vázquez J.A., Menduíña A., Durán A.I., Nogueira M., Fernández-Compás A., Pérez-Martín R.I., Amado I.R. (2019). Production of valuable compounds and bioactive metabolites from by-products of fish discards using chemical processing, enzymatic hydrolysis, and bacterial fermentation. Mar. Drugs.

[27] Vázquez J.A., Fraguas J., Mirón J., Valcarcel J., Pérez-Martín R.I., Antelo L.T. (2020). Valorisation of fish discards assisted by enzymatic hydrolysis and microbial bioconversion: Lab and pilot plant studies and preliminary sustainability evaluation. J. Clean. Prod..

[28] Bernfeld P. (1951). Enzymes of starch degradation and synthesis. Adv. Enzymol..

[29] Lowry O.H., Rosebrough N.J., Farr A.L., Randall R.J. (1951). Protein measurement with the folin phenol reagent. J. Biol. Chem..

[30] Dubois M., Gilles K.A., Hamilton J.K., Rebers P.A., Smith F. (1956). Colorimetric method for determination of sugars and related substances. Anal. Chem..

[31] Vázquez J.A., Lorenzo J.M., Fuciños P., Franco D. (2012). Evaluation of non-linear equations to model different animal growths with mono and bi-sigmoid profiles. J. Theor. Biol..

[32] Vázquez J.A., Caprioni R., Nogueira M., Menduiña A., Ramos P., Pérez-Martín R.I. (2016). Valorisation of effluents obtained from chemical and enzymatic chitin production of *Illex argentinus* pen by-products as nutrient supplements for various bacterial fermentations. Biochem. Eng. J..

[33] Vázquez J.A., González M.P., Murado M.A. (2004). A new marine medium. Use of the different fish peptones and comparative study of the growth of selected species of marine bacteria. Enzyme Microb. Technol..

[34] Vázquez J.A., Docasal S.F., Mirón J., González M.P., Murado M.A. (2006). Proteases production by two Vibrio species on residuals marine media. J. Ind. Microbiol. Biotechnol..

[35] Lapeña D., Vuoristo K.S., Kosa G., Horn S.J., Eijsink V.G.H. (2018). Comparative assessment of enzymatic hydrolysis for valorization of different protein-rich industrial byproducts. J. Agric. Food Chem..

[36] Tan Y., Chang S.K.C., Meng S. (2019). Comparing the kinetics of the hydrolysis of by-product from channel catfish (*Ictalurus punctatus*) fillet processing by eight proteases. LWT.

[37] Vázquez J.A., González M.P., Murado M.A. (2004). Peptones from autohydrolysed fish viscera for nisin and pediocin production. J. Biotechnol..

[38] Castro-Ceseña A.B., Sánchez-Saavedra M.P., Márquez-Rocha F.J. (2012). Characterisation and partial purification of proteolytic enzymes from sardine by-products to obtain concentrated hydrolysates. Food Chem..

[39] Derouiche B.M.H., Guadix E.M., Guadix A., Gargouri M., Espejo-Carpio F.J. (2019). Valorisation of tuna viscera by endogenous enzymatic treatment. Int. J. Food Sci. Technol..

[40] Deraz S.F., El-Fawal G.F., Abd-Ellatif S.A., Khalil A.A. (2011). Autohydrolysed *Tilapia nilotica* fish viscera as a peptone source in bacteriocin production. Indian J. Microbiol..

[41] Vázquez J.A., Amado I.R., Sotelo C.G., Sanz N., Pérez-Martín R.I., Valcarcel J. (2020). Production, characterization, and bioactivity of fish protein hydrolysates from aquaculture turbot (*Scophthalmus maximus*) wastes. Biomolecules.

[42] Halim N.R.A., Yusof H.M., Sarbon N.M. (2016). Functional and bioactive properties of fish protein hydolysates and peptides: A comprehensive review. Trends Food Sci. Technol..

[43] Fan B., Sun J., Dong P., Xue C., Mao X. (2017). Conversion of turbot skin wastes into valuable functional substances with an eco-friendly fermentation technology. J. Clean. Prod..

[44] Aspmo S.I., Horn S.J., Eijsink V.G.H. (2005). Use of hydrolysates from Atlantic cod (*Gadus morhua* L.) viscera as a complex nitrogen source for lactic acid bacteria. FEMS Microbiol. Lett..

[45] Safari R., Saravi H.N., Pourgholam R., Motalebi A.A., Ghoroghi A. (2011). Use of hydrolysates from silver carp (*Hypophthalmichthys molitrix*) head as peptone for *Vibrio anguillarum* and optimization using response surface method (RSM). J. Aquat. Food Prod. Technol..

[46] Strathe A.B., Danfær A., Sørensen H., Kebreab E. (2010). A multi level non linear mixed-effects approach to model growth in pigs. J. Anim. Sci..

[47] Franco D., Rois D., Vázquez J.A., Purriños L., González R., Lorenzo J.M. (2012). Breed effect between Mos rooster (Galician indigenous breed) and SassoT-44 line and finishing feed effect of commercial fodder or corn. Poult. Sci..

[48] Chhabra A.T., Carter W.H., Linton R.H., Cousin M.A. (2002). A predictive model that evaluates the effect of growth conditions on the thermal resistance of *Listeria monocytogenes*. Int. J. Food Microbiol..

[49] Vázquez J.A., Montemayor M.I., Fraguas J., Murado M.A. (2009). High production of hyaluronic and lactic acids by *Streptococcus zooepidemicus* in fed-batch cultures using commercial and marine peptones from fishing by-products. Biochem. Eng. J..

[50] Rutledge R.G., Stewart D. (2008). A kinetic-based sigmoidal model for the polymerase chain reaction and its application to high-capacity absolute quantitative real-time PCR. BMC Biotechnol..

