# Supplementary material for: Production of Marine Probiotic Bacteria in a Cost-Effective Marine Media Based on Peptones Obtained from Discarded Fish By-Products
Source: Microorganisms. 2020 Jul 26;8(8):1121. doi: 10.3390/microorganisms8081121 (PMC7464406; doi:10.3390/microorganisms8081121)
Supplement: Supplementary file 1 [file microorganisms-08-01121-s001.pdf]

Supplementary Material

Production of marine probiotic bacteria in a cost-effective marine media based on peptones obtained from fish discards by-products.

José Antonio Vázquez[1,2*], Ana I. Durán[1,2], Araceli Menduíña[1,2], Margarita Nogueira[1,2], Joana Antunes[3], Ana Cristina Freitas[3] and Ana María Gomes[3]

1 Grupo de Biotecnología y Bioprocesos Marinos, Instituto de Investigaciones Marinas (IIM-CSIC), C/ Eduardo Cabello, 6, CP 36208, Vigo, Galicia – España. jvazquez@iim.csic.es (J.A.V.); anais@iim.csic.es (A.I.D.); araceli@iim.csic.es (A.M.); marga@iim.csic.es (M.N.); jvalcarcel@iim.csic.es (J.V.)

2 Laboratorio de Reciclado y Valorización de Materiales Residuales (REVAL), Instituto de Investigaciones Marinas (IIM-CSIC), C/ Eduardo Cabello, 6, CP 36208, Vigo, Galicia – España;

3 Universidade Católica Portuguesa, CBQF - Centro de Biotecnologia e Química Fina - Laboratório Associado, Escola Superior de Biotecnologia, Rua Diogo Botelho 1327, 4169-005 Porto, Portugal. amgomes@porto.ucp.pt (A.M.G.); jantunes@porto.ucp.pt (J.A.); afreitas@porto.ucp.pt (A.C.F.)

* Correspondence: jvazquez@iim.csic.es

Table S1. Basic biochemical composition of fish peptones (mean values±confidence intervals). Pr: Total soluble protein; RS: Reducing sugars; TS: Total sugars. Sk, He, and Wh mean skin, head and whole individual. BW: blue whiting, RS: red scorpionfish, Ma: mackerel; Po: pouting; Gu: gurnard; Gr: grenadier; Me: megrim; Ha: hake; Bo: boardfish and AHM: Atlantic horse mackerel.

Fish Peptones	Pr (g/L)	RS (g/L)	TS (g/L)	Fish Peptones	Pr (g/L)	RS (g/L)	TS (g/L)
**Sk_BW**	44.8±3.1	0.13±0.02	0.65±0.06	**He_Gr**	29.4±0.7	0.19±0.05	0.83±0.04
**Sk_RS**	39.7±3.6	0.17±0.04	0.74±0.16	**He_Me**	34.5±1.6	0.20±0.02	0.62±0.06
**Sk_Ma**	35.7±2.4	0.42±0.18	1.61±0.50	**He_Ha**	29.5±0.3	0.24±0.09	0.79±0.08
**Sk_Po**	42.7±4.0	0.09±0.01	0.45±0.09	**He_Bo**	29.1±4.8	0.16±0.10	0.87±0.12
**Sk_Gu**	39.7±2.7	0.27±0.03	0.92±0.07	**He_AHM**	27.7±0.9	0.36±0.06	1.06±0.07
**Sk_Gr**	42.2±2.0	0.31±0.04	0.73±0.01	**Wh_BW**	47.8±4.8	0.42±0.09	1.20±0.07
**Sk_Me**	40.4±3.1	0.11±0.09	0.50±0.02	**Wh_RS**	36.8±1.6	0.12±0.00	0.60±0.02
**Sk_Ha**	33.1±0.5	0.13±0.05	0.59±0.02	**Wh_Ma**	36.4±0.7	0.11±0.00	0.74±0.31
**Sk_Bo**	34.2±0.5	0.45±0.12	1.15±0.05	**Wh_Po**	44.3±2.3	0.14±0.02	0.79±0.05
**Sk_AHM**	38.4±0.1	0.20±0.03	0.70±0.01	**Wh_Gu**	41.1±5.4	0.24±0.03	0.92±0.00
**He_BW**	34.7±3.4	0.31±0.08	0.79±0.01	**Wh_Gr**	47.1±1.1	0.09±0.01	0.50±0.02
**He_RS**	39.2±1.5	0.32±0.09	1.25±0.06	**Wh_Me**	53.9±5.1	0.28±0.12	1.06±0.25
**He_Ma**	31.4±2.8	0.25±0.07	0.89±0.04	**Wh_Ha**	36.5±1.7	0.16±0.02	0.72±0.08
**He_Po**	39.6±0.4	0.21±0.07	0.68±0.05	**Wh_Bo**	39.3±1.9	0.41±0.06	1.31±0.37
**He_Gu**	33.5±5.8	0.32±0.06	1.45±0.26	**Wh_AHM**	47.6±3.2	0.33±0.10	1.40±0.23

Table S2. Composition of culture media for marine probiotic bacteria (g/L).

INGREDIENTS	Alternative media	MM
**Ferric citrate**	-	0.10
**Sodium chloride**	-	19.45
**Magnesium chloride**	-	5.90
**Sodium sulphate**	-	3.24
**Calcium chloride**	-	1.80
**Potassium chloride**	-	0.55
**Sodium bicarbonate**	-	0.16
**Potassium bromide**	-	0.08
**Strontium chloride**	-	34.0 mg
**Boric acid**	-	22.0 mg
**Sodium silicate**	-	4.0 mg
**Sodium fluoride**	-	2.4 mg
**Ammonium nitrate**	-	1.6 mg
**Disodium phosphate**	-	8.0 mg
**Yeast extract**	1.0	1.0
**Peptone**	-	5.0
**Fish peptone as protein-Lowry**	2.6	-
**Sea water (L)***	1.0	-
**Distilled water (L)****	-	1.0

Alternative media: formulated with the marine peptones obtained from fish discarded.

*Volume of filtrated and sterilized sea water needed for residual media preparation.
**Volume of distilled water needed for commercial medium preparation.

Table S3. Numerical values and confidence intervals for parameters obtained from experimental data of *Pseudomonas fluorescens* (Pf) growths modelled by logistic equation (1-4). R^2 is the determination coefficient among experimental and predicted data. The production yields ($Y_{X/Pr}$ and $Y_{G/Pr}$) are also shown. NS: not significant. MM1 and MM2 were the controls kinetics in commercial marine medium (MM) performed for each set of cultures. Consistency of fittings were also determined (p-value from F-Fisher test).

Biomass (X)

	Sk_BW	Sk_RS	Sk_Ma	Sk_Po	Sk_Gu	Sk_Gr	Sk_Me	Sk_Ha	Sk_Bo	Sk_AHM	He_BW	He_RS	He_Ma	He_Po	He_Gu	MM1
X_m	1.17±0.03	0.99±0.06	1.11±0.05	1.17±0.04	1.13±0.07	1.11±0.08	1.06±0.15	1.07±0.04	1.11±0.07	1.14±0.05	1.12±0.07	1.13±0.05	1.03±0.04	1.14±0.08	1.10±0.03	1.06±0.06
v_m	0.12±0.01	0.10±0.03	0.11±0.02	0.11±0.02	0.10±0.02	0.09±0.03	0.06±0.02	0.11±0.02	0.11±0.03	0.11±0.02	0.10±0.02	0.11±0.02	0.10±0.02	0.09±0.03	0.11±0.01	0.13±0.04
λ_X	6.74±0.51	5.93±1.30	6.10±1.03	6.44±0.85	5.86±1.31	5.33±1.66	3.76±2.68	5.96±0.88	6.40±1.27	6.28±0.99	5.91±1.32	6.01±0.99	6.04±0.95	5.24±1.57	6.26±0.67	7.01±1.35
μ_X	0.42±0.05	0.42±0.12	0.39±0.08	0.39±0.07	0.35±0.09	0.34±0.10	0.22±0.08	0.42±0.08	0.39±0.10	0.37±0.07	0.36±0.09	0.39±0.08	0.39±0.07	0.34±0.10	0.39±0.05	0.51±0.17
τ_X	11.5±0.3	10.7±0.8	11.2±0.6	11.6±0.5	11.6±0.8	11.2±1.1	13.0±2.3	10.8±0.5	11.6±0.8	11.6±0.6	11.4±0.8	11.2±0.6	11.2±0.6	11.1±1.0	11.5±0.4	11.0±0.8
t_{mX}	16.3±0.7	15.5±1.7	16.4±1.4	16.8±1.2	17.3±1.9	17.1±2.4	22.3±5.2	15.6±1.2	16.7±1.8	17.0±1.4	17.0±1.9	16.4±2.4	26.4±1.3	16.9±2.3	16.7±2.4	14.9±1.7
$Y_{X/Pr}$	2.177	1.180	1.362	2.167	1.321	1.364	2.081	1.311	1.317	1.286	1.306	1.585	1.140	1.381	1.324	1.531
R^2	0.999	0.994	0.997	0.998	0.995	0.993	0.985	0.998	0.995	0.997	0.995	0.997	0.997	0.993	0.999	0.994
p-value	<0.005	<0.005	<0.005	<0.005	<0.005	<0.005	<0.005	<0.005	<0.005	<0.005	<0.005	<0.005	<0.005	<0.005	<0.005	<0.005

Cells (G)

	Sk_BW	Sk_RS	Sk_Ma	Sk_Po	Sk_Gu	Sk_Gr	Sk_Me	Sk_Ha	Sk_Bo	Sk_AHM	He_BW	He_RS	He_Ma	He_Po	He_Gu	MM1
G_m	12.3±1.4	13.3±2.0	12.5±2.1	11.8±1.8	12.5±1.4	13.5±1.7	13.6±2.2	13.2±1.2	13.2±1.6	13.3±1.5	13.3±0.9	13.4±0.7	13.7±1.5	12.7±1.6	13.6±0.7	12.7±1.4
v_G	0.91±0.37	0.91±0.40	1.05±0.68	0.83±0.41	1.08±0.48	0.85±0.31	0.75±0.32	0.91±0.27	1.05±0.47	0.89±0.28	1.09±0.27	1.17±0.25	0.92±0.29	1.01±0.45	1.19±0.26	0.87±0.31
λ_G	2.72 (NS)	2.79 (NS)	3.71 (NS)	2.70 (NS)	3.26±2.74	1.97 (NS)	0.66 (NS)	2.03 (NS)	3.27±2.97	4.24±2.43	3.58±1.59	3.24±1.33	4.24±2.43	3.72±2.97	3.24±1.33	2.88±2.67
μ_G	0.30±0.14	0.27±0.15	0.34±0.24	0.28±0.16	0.35±0.17	0.25±0.11	0.22±0.11	0.27±0.09	0.32±0.16	0.27±0.10	0.33±0.09	0.35±0.08	0.27±0.10	0.32±0.16	0.35±0.08	0.28±0.11
τ_G	9.44±1.78	10.1±2.32	9.67±2.48	9.85±2.36	9.06±1.61	9.95±2.02	9.75±2.79	9.34±1.45	10.0±1.8	11.7±1.7	9.70±0.96	8.94±0.78	11.7±1.7	10.0±1.8	8.94±0.78	10.2±1.8
t_{mG}	16.2±4.1	17.4±5.3	15.6±5.6	17.0±5.4	14.9±3.6	17.9±0.8	18.8±0.8	16.7±3.4	16.3±4.2	19.1±3.9	15.8±2.2	14.6±1.7	19.1±3.9	16.3±4.2	14.6±1.7	17.4±4.0
$Y_{G/Pr}$	24.50	15.93	17.21	23.85	15.41	16.94	27.82	16.63	17.53	15.61	16.04	19.09	16.07	16.31	16.41	18.85
R^2	0.978	0.969	0.954	0.968	0.979	0.980	0.966	0.987	0.978	0.987	0.994	0.995	0.987	0.978	0.995	0.983
p-value	<0.005	<0.005	<0.005	<0.005	<0.005	<0.005	<0.005	<0.005	<0.005	<0.005	<0.005	<0.005	<0.005	<0.005	<0.005	<0.005

Table S4. continuation of Table S3.

Biomass (X)																
	**He_Gr**	**He_Bo**	**He_Ha**	**He_AHM**	**He_Me**	**Wh_BW**	**Wh_RS**	**Wh_Ma**	**Wh_Po**	**Wh_Gu**	**Wh_Gr**	**Wh_Bo**	**Wh_Ha**	**Wh_Me**	**Wh_AHM**	**MM2**
X_m	0.90±0.04	0.87±0.06	0.99±0.05	1.06±0.15	1.10±0.08	0.91±0.04	1.04±0.04	0.97±0.03	0.99±0.05	0.91±0.16	1.02±0.06	0.94±0.07	0.93±0.03	0.93±0.05	1.10±0.04	1.03±0.06
v_m	0.09±0.02	0.09±0.03	0.10±0.02	0.06±0.02	0.09±0.02	0.08±0.01	0.10±0.01	0.10±0.01	0.09±0.02	0.08±0.05	0.09±0.02	0.07±0.02	0.09±0.01	0.08±0.02	0.11±0.02	0.12±0.01
λ_X	5.99±1.05	6.48±1.61	6.43±1.02	3.76±2.68	4.99±1.58	5.64±0.94	6.39±0.74	6.17±0.67	6.01±1.07	5.64±3.96	5.51±1.20	5.51±1.51	5.49±0.63	5.25±1.12	6.26±0.76	5.01±1.23
μ_X	0.42±0.10	0.41±0.14	0.39±0.08	0.22±0.08	0.32±0.09	0.35±0.06	0.40±0.06	0.40±0.05	0.34±0.07	0.35±0.26	0.36±0.08	0.31±0.08	0.36±0.04	0.35±0.07	0.39±0.04	0.46±0.06
τ_X	10.8±0.6	11.4±1.0	11.4±0.6	13.0±2.3	11.2±1.0	11.3±0.6	11.4±0.5	11.2±0.4	11.9±0.7	11.3±2.5	11.1±0.8	11.9±1.0	11.0±0.4	11.0±0.7	11.4±0.3	11.6±0.8
t_{mX}	15.5±1.4	16.3±2.2	16.6±1.4	22.3±5.2	17.4±2.3	17.0±1.4	16.5±1.0	16.3±0.9	17.7±1.6	17.0±5.7	16.8±1.7	18.4±2.3	16.5±0.9	16.8±1.6	16.5±0.8	15.1±1.9
$Y_{X/Pr}$	1.054	1.109	1.170	2.081	1.317	1.087	1.211	1.100	1.180	1.189	1.224	1.149	1.325	1.335	1.328	1.970
R^2	0.996	0.992	0.997	0.985	0.994	0.998	0.998	0.999	0.997	0.998	0.996	0.994	0.999	0.997	0.999	0.996
p-value	<0.005	<0.005	<0.005	<0.005	<0.005	<0.005	<0.005	<0.005	<0.005	<0.005	<0.005	<0.005	<0.005	<0.005	<0.005	<0.005

Cells (G)																
	**He_Gr**	**He_Bo**	**He_Ha**	**He_AHM**	**He_Me**	**Wh_BW**	**Wh_RS**	**Wh_Ma**	**Wh_Po**	**Wh_Gu**	**Wh_Gr**	**Wh_Bo**	**Wh_Ha**	**Wh_Me**	**Wh_AHM**	**MM**
G_m	12.4±1.4	13.5±1.2	13.0±0.8	13.2±1.7	12.6±1.5	13.6±0.9	13.3±1.0	13.1±1.1	13.2±1.3	13.3±1.1	13.1±1.2	13.1±1.4	13.4±1.2	13.1±1.2	13.2±0.5	13.5±1.0
v_G	0.85±0.30	0.92±0.28	1.06±0.26	0.79±0.31	0.82±0.27	1.18±0.31	1.06±0.29	0.98±0.27	1.00±0.32	1.11±0.34	0.99±0.33	1.08±0.42	1.07±0.35	0.92±0.28	1.30±0.21	1.12±0.30
λ_G	2.88±2.67	2.03±2.01	3.58±1.59	0.93±0.92	3.29±2.60	3.74±1.63	4.07±1.81	4.66±1.89	3.99±2.22	3.53±1.94	2.95±2.31	3.60±2.54	3.62±2.15	2.27±2.26	4.22±0.91	3.88±1.73
μ_G	0.28±0.11	0.27±0.09	0.33±0.09	0.24±0.11	0.26±0.10	0.35±0.12	0.32±0.10	0.30±0.09	0.30±0.11	0.33±0.11	0.30±0.11	0.33±0.14	0.32±0.12	0.28±0.10	0.39±0.07	0.33±0.10
τ_G	10.2±1.8	9.34±1.50	9.70±0.96	9.24±2.18	11.2±1.8	9.48±1.15	10.4±1.1	11.4±1.3	10.6±1.4	9.54±1.2	9.55±1.43	9.70±1.54	9.86±1.32	9.34±1.41	9.32±0.52	9.91±1.05
t_{mG}	17.4±4.0	16.7±3.4	15.8±2.2	17.6±5.2	18.8±4.2	15.2±2.2	16.7±2.6	18.1±2.9	17.1±3.3	15.5±2.7	16.1±3.3	15.8±3.5	16.1±3.0	16.4±3.3	14.4±1.2	15.9±2.4
$Y_{G/Pr}$	15.29	16.97	15.72	26.78	15.52	16.57	15.80	15.36	16.55	16.36	15.98	16.89	19.48	18.85	16.22	24.21
R^2	0.983	0.987	0.994	0.974	0.985	0.993	0.992	0.991	0.988	0.990	0.986	0.983	0.988	0.987	0.998	0.992
p-value	<0.005	<0.005	<0.005	<0.005	<0.005	<0.005	<0.005	<0.005	<0.005	<0.005	<0.005	<0.005	<0.005	<0.005	<0.005	<0.005

Table S5. Costs of production of biomass and viable cells in the commercial marine medium and in the media formulated with fish peptones and seawater. Sk: skin+bones, He: head and Wh: whole individuals. BW: blue whiting, RS: red scorpionfish, Ma: mackerel, Po: pouting, Gu: gurnard, Gr: granadier, Me: megrim, Ha: hake, Bo: boardfish and AHM: Atlantic horse mackerel. CM: commercial media, AM: alternative media.

		***Phaeobacter* sp.**		***P. fluorescens***	
	**Culture media**	**Biomass (€/g)**	**Cells (€/cell)**	**Biomass (€/g)**	**Cells (€/cell)**
**MM1**	CM	8.96±0.44	0.947±0.208	8.54±0.48	0.713±0.079
**MM2**	CM	9.53±1.00	0.906±0.198	8.79±0.51	0.670±0.050
**Sk_BW**	AM formulated with Sk_BW peptones	0.095±0.004	0.010±0.000	0.077±0.002	0.007±0.001
**Sk_RS**	AM formulated with Sk_RS peptones	0.096±0.004	0.009±0.001	0.091±0.002	0.007±0.001
**Sk_Ma**	AM formulated with Sk_Ma peptones	0.094±0.004	0.009±0.001	0.081±0.002	0.008±0.001
**Sk_Po**	AM formulated with Sk_Po peptones	0.095±0.005	0.009±0.001	0.077±0.003	0.007±0.001
**Sk_Gu**	AM formulated with Sk_Gu peptones	0.086±0.003	0.008±0.001	0.080±0.005	0.007±0.001
**Sk_Gr**	AM formulated with Sk_Gr peptones	0.090±0.005	0.009±0.001	0.081±0.005	0.007±0.001
**Sk_Me**	AM formulated with Sk_Me peptones	0.100±0.009	0.010±0.001	0.085±0.012	0.007±0.000
**Sk_Ha**	AM formulated with Sk_Ha peptones	0.099±0.009	0.009±0.001	0.084±0.004	0.007±0.000
**Sk_Bo**	AM formulated with Sk_Bo peptones	0.074±0.009	0.008±0.001	0.081±0.005	0.007±0.001
**Sk_AHM**	AM formulated with Sk_AHM peptones	0.125±0.024	0.010±0.000	0.079±0.006	0.007±0.001
**He_BW**	AM formulated with He_BW peptones	0.103±0.011	0.008±0.001	0.080±0.007	0.007±0.000
**He_RS**	AM formulated with He_RS peptones	0.117±0.018	0.010±0.000	0.080±0.006	0.007±0.001
**He_Ma**	AM formulated with He_Ma peptones	0.115±0.019	0.010±0.001	0.087±0.003	0.007±0.001
**He_Po**	AM formulated with He_Po peptones	0.098±0.014	0.010±0.001	0.079±0.006	0.007±0.001
**He_Gu**	AM formulated with He_Gu peptones	0.078±0.004	0.009±0.001	0.082±0.005	0.007±0.000
**He_Gr**	AM formulated with He_Gr peptones	0.108±0.014	0.009±0.001	0.100±0.007	0.007±0.001
**He_Me**	AM formulated with He_Me peptones	0.096±0.009	0.009±0.001	0.082±0.006	0.007±0.001
**He_Ha**	AM formulated with He_Ha peptones	0.108±0.013	0.009±0.001	0.091±0.005	0.007±0.000
**He_Bo**	AM formulated with He_Bo peptones	0.118±0.011	0.010±0.001	0.103±0.006	0.007±0.000
**He_AHM**	AM formulated with He_AHM peptones	0.081±0.006	0.008±0.001	0.085±0.012	0.007±0.000
**Wh_BW**	AM formulated with Wh_BW peptones	0.106±0.006	0.010±0.001	0.099±0.011	0.007±0.000
**Wh_RS**	AM formulated with Wh_RS peptones	0.130±0.038	0.011±0.002	0.087±0.006	0.007±0.000
**Wh_Ma**	AM formulated with Wh_Ma peptones	0.106±0.014	0.008±0.001	0.093±0.007	0.007±0.000
**Wh_Po**	AM formulated with Wh_Po peptones	0.132±0.037	0.010±0.001	0.091±0.005	0.007±0.001
**Wh_Gu**	AM formulated with Wh_Gu peptones	0.130±0.032	0.009±0.001	0.099±0.011	0.007±0.000
**Wh_Gr**	AM formulated with Wh_Gr peptones	0.122±0.026	0.009±0.001	0.088±0.005	0.007±0.000
**Wh_Me**	AM formulated with Wh_Me peptones	0.127±0.013	0.010±0.000	0.097±0.006	0.007±0.000
**Wh_Ha**	AM formulated with Wh_Ha peptones	0.132±0.010	0.011±0.001	0.097±0.004	0.007±0.001
**Wh_Bo**	AM formulated with Wh_Bo peptones	0.079±0.014	0.008±0.001	0.096±0.005	0.007±0.001
**Wh_AHM**	AM formulated with Wh_AHM peptones	0.111±0.005	0.010±0.001	0.082±0.003	0.007±0.000
